# Sex blind: bridging the gap between drug exposure and sex-related gene expression in *Danio rerio* using next-generation sequencing (NGS) data and a literature review to find the missing links in pharmaceutical and environmental toxicology studies

**DOI:** 10.3389/ftox.2023.1187302

**Published:** 2023-06-16

**Authors:** Alex C. King, Armin K. Zenker

**Affiliations:** University of Applied Sciences and Arts North-Western Switzerland (FHNW), Muttenz, Switzerland

**Keywords:** sex-variation, gene expression, zebrafish, drug influence, human health

## Abstract

The sex of both humans and *Danio rerio* has previously been shown to affect the way individuals respond to drug exposure. Genes which allow identification of sex in juvenile zebrafish show potential to reveal these confounding variables between sex in toxicological and preclinical trials but the link between these is so far missing. These sex-specific, early expressed genes where expression is not altered by drug exposure must be carefully selected for this purpose. We aimed to discover genes which can be used in pharmaceutical trials and environmental toxicology studies to uncover sex-related variations in gene expression with drug application using the model organism *Danio rerio*. Previously published early sex determining genes from King et al. were evaluated as well as additional genes selected from our zebrafish Next-generation sequencing (NGS) data which are known from previously published works not to be susceptible to changes in expression with drug exposure. NGS revealed a further ten female-specific genes (vtg1, cyp17a1, cyp19a1a, igf3, ftz-f1, gdf9, foxl2a, Nr0b1, ipo4, lhcgr) and five male related candidate genes (FKBP5, apobb1, hbaa1, dmrt1, spata6) which are also expressed in juvenile zebrafish, 28 days post fertilisation (dpf). Following this, a literature review was performed to classify which of these early-expressed sex specific genes are already known to be affected by drug exposure in order to determine candidate genes to be used in pharmaceutical trials or environmental toxicology testing studies. Discovery of these early sex-determining genes in *Danio rerio* will allow identification of sex-related responses to drug testing to improve sex-specific healthcare and the medical treatment of human patients.

## 1 Introduction

Today*, Danio rerio* (Zebrafish) are popular model organisms for use in many scientific fields of study. The characteristics of embryonic development in zebrafish including their rapid growth phase, high fecundity and transparency make them ideal as a model organism (Grunwald and Eisen, 2002; Segner, 2009). Zebrafish are frequently used as a pharmacological tool in drug discovery (Goldsmith, 2004; Zon and Peterson, 2005), in environmental risk assessment studies and to aid understanding of human form, function and mechanisms of disease (Nagabhushana and Mishra, 2016; Chen et al., 2017).

Adult, sexually mature zebrafish are used in the 21-day Fish Assay, a short-term screening for oestrogenic and androgenic activity, and aromatase inhibition (OECD, 2009). In these tests, sex can be easily determined both morphologically and genetically. However, during both Fish Embryo Acute Toxicity (FET) tests (OECD, 2013), which measure acute toxicity and multi-effect detecting Fish Early-Life Stage (FELS) toxicity test (OECD, 2010), the sex of the embryos is indistinguishable (Kimmel et al., 1995). The importance of knowing the sex of the individual being tested comes as sex-specific differences to drug exposure are revealed (Kling et al., 2008; Dlugos et al., 2011). Puzzling results from FET and FELS tests are often overlooked as being caused by differing effects of various drugs on sex (Holdcroft, 2007). Males and females react in a different way to exposure but so far FET and FELS tests fail to adequately link sex-related variations in gene expression to drug exposure (Liu et al., 2021; Fisher et al., 2022). Knowledge of sex at juvenile stages during these tests is fundamental to prevent false or unclear results being presented due to confounding sex variables which mask differences to drug application. By revealing sex during these early-stage tests we can discover the effect of the drugs in later life as well as the sensitivity of male and females to each drug. FELS and FET tests have several advantages when compared to the 21-day fish assay, they both use younger, smaller fish which means that fish are more susceptible to the effects of drugs (Vasconcelos et al., 2010; Mohammed, 2011), as well as being cheaper to use than adult fish. The 3Rs, reduce, refine, replace philosophy aims to decrease the use of animals in laboratory tests and minimise their pain by improving their welfare standards; this theory arguable points to use of juvenile fish over adults (Flecknell, 2002; Leconte et al., 2011).

In mammals and fish, there is considerable evidence that sex creates variations in susceptibility and response to exposure of drugs (Gartlehner et al., 2010). Use of a certain pharmaceuticals or exposure to specific chemicals results in changes to hormone levels as well as altering the regulation of specific sex differentiation genes (Kokane and Perrotti, 2020). Pharmaceuticals with low concentrations of active progesterone (P4) or norgestrel (NGT) as well as those containing substances which alter steroidogenic genes change the balance of sex hormones, namely, oestrogens and androgens, in juvenile zebrafish. These are thought to consequently affect the expression level of sex-determining genes (Sox9a, gapdhs, atp1b1a, cyp26b1, gyg1a, rdh10b, pdia, KPNA2, ccnb1, ctsla, Chr4, bmp15 and zbp3) (King et al., 2020); therefore, disrupting sex differentiation in zebrafish.

The action of some drugs (anti-inflammatories) is driven by the oestrogen pathway, thereby making females more susceptible to these drugs than males (Houben et al., 2020; Kokane and Perrotti, 2020). Opposingly oestrogen can have an inhibitory effect on the way a female liver breaks down medication (Osman, 2003; Martín-Millán and Castañeda, 2013). Studies have previously shown that developmental exposure of zebrafish to 17-Alpha-ethinyloestradiol (EE2) increases adult anxiety and shoal cohesion as well as changing gene expression, these outcomes were found to differ significantly with sex (Volkova et al., 2015). Other studies have also shown that application of P4 results in a female sex-biased population; whereas NGT results in a male dominated population (Liang et al., 2015). P4 is mostly found in human and animal excretion and is an essential regulator for the growth and maturation of oocytes. Synthetic progestins are often active ingredients of human pharmaceuticals, including oral and implant contraceptives (Kejuan et al., 2007).

Awareness of the differing responses that drug exposure has on male and females is important and can be life changing. Pharmacokinetics and pharmacodynamics as well as differences in genetic make-up, gene expression and hormone regulation of drug metabolising enzymes and transporters, body weight, height, body surface area and total water body content are significantly different in men and women (Soldin and Mattison, 2009; Plank-Bazinet et al., 2016). These factors create sex-related variations in the absorption, distribution, metabolism, and excretion of drugs, which in turn affects the drug efficacy and creates variations in adverse reactions. This points to evidence suggesting that personalised medicine to cater for differences between males and female would be beneficial for improving reactions to drug response and for understanding sex-based disease susceptibilities (U.S. Government Accountability Office, 2001; Nicolson et al., 2010; Lyubimov et al., 2012; Ridker et al., 2015). Previous trials with mice show that the dosage of sodium amytal for males can be half that which is effective to anesthetise females (Yang and Li, 2012). CYP3A4-substrate drugs, including, cyclosporine, erythromycin, tirilazad, verapamil, nifedipine, diazepam and alfentanil, take longer to clear in women than men, even after taking into consideration sex-specific physiological factors (Franconi et al., 2007; Greenblatt and Von Moltke, 2008). Additionally, volunteers for drug tests are normally male, non-smokers aged between 25–35. Therefore, there is a gross underestimation of female susceptibility to drugs because of this (Kim et al., 2010; McCarthy et al., 2017).

The main purpose of this study is to identify whether sex specific genes which are expressed during juvenile stages of zebrafish development are known to be affected by drug exposure. For this purpose, we used transcript data from NGS of zebrafish to select additional appropriate novel, early expressed, sex-specific genetic markers, thereby, further unravelling the puzzle of sex-differentiating genes which are expressed during juvenile development. The carefully selected juvenile-expressed genes shown in this paper were categorised based on a literature review of results from previously published studies in order to indicate any sex-related differences in expression of these genes due to interference from drug exposure. Our research will aid future zebrafish studies as well as helping to uncover the confounding variable of sex during drug testing. This will allow underlying, masked sex-related differences in response to pharmaceuticals to be highlighted. In turn, an advancement in sex-specific drug development can be achieved by providing knowledge regarding sex variations in which to build and develop appropriate medication including providing safe doses of drugs for both males and females.

## 2 Experimental procedures

### 2.1 RNA sequencing using NGS

NGS RNA sequences were produced from two 28-day-old zebrafish (which we classified as juvenile in this paper), two adult male and two adult female zebrafish using whole-body tissue samples. Juveniles were tested to indicate whether sex was genetically visible at this stage of development. RNA quality testing and NGS were performed at the Biocentre under the supervision of Philippe Demougin (Life Sciences Training Facility (LSTF), Basel), according to Illumina’s standard protocol for RNA sequencing (Illumina Inc., San Diego, USA, Cat. # RS-100-0801). The NGS transcript data of the sampled zebrafish were compared to identify possible genes that contribute to sex determination as well as those which are expressed early in juvenile development, 28dpf (King et al., 2020). All data analyses steps were carried out using the Galaxy server (usegalaxy.org) (Jalili et al., 2020). Data from transcript sequencing was mapped to the GRCz11 reference genome (GCA_000002035.4) using HISAT2 (Galaxy Version 2.1.0+galaxy4) (Kim et al., 2019). Transcripts were assembled and merged from the mapped reads with StringTie (Galaxy Version 1.3.4) (Pertea et al., 2015). After determining gene expression with featureCount (Galaxy Version 1.6.3+galaxy2), the NGS data was normalised and differential gene expression analysis of juvenile, male and female zebrafish was carried out using DESeq2 (Galaxy Version 2.11.40.2) (Love et al., 2014; King et al., 2020). Normalisation was based on the ‘median of ratios’ - the counts divided by sample-specific size factors determined by median ratio of gene counts relative to geometric mean per gene, to account for sequencing depth and RNA composition. This allowed us to produce a table of gene expression which highlighted genes with the highest transcript count difference between males and females. From this data, genes expressed at juvenile-stage (28dpf) which were also expressed highly in male or female samples were selected as the best gene candidates for indicating early sex identity of zebrafish. Four male (Sox9a, Gapdhs, atp1b1a and cyp26b1) and nine female genes (gyg1a, rdh10b, pdia, KPNA2, ccnb1, ctsla, Chr4, bmp15 and zbp3) which were previously identified as an early sex-determining marker in zebrafish (King et al., 2020) were used within a literature search to access previous information as to whether their level of expression was known to be influenced by certain drugs. In this study a further ten female and five male genes, which could be used in early sex-determination were selected from NGS data. A literature search was also carried out on these genes to discover if they were known to be affected by drug usage. Based on published literature from ScienceDirect in early 2021, we obtained information previous studies from the past 20 years by searching for the key terms; including the gene name, drug or pharmaceutical exposure, sex, or gender influence. Then, we eliminated irrelevant literature by reading the titles and abstracts and supplemented our literature database by reading the references of the selected studies. We used the selected literature to inform whether genes highlighted from our NGS data as early expressed within juveniles could be candidates in drug trials. Finally, we grouped early expressed genes from NGS data based on the literature search into three categories. If the study was shown to affect the expression of the selected genes, then they were deemed not good to use in pharmaceutical trials. Conversely, if it was not known from previous research that drugs affect the expression of a certain gene, it was included as a potential candidate for revealing how sex response differs with drug exposure and further investigation of its use in this area is necessary in future research. Statistics were not performed due to the small sampling size but gene expression tables were produced to indicate genes with high variation between males and females.

### 2.2 Statement on experimental protocols

NGS data was collected from 28-day-old and adult zebrafish, which had already been euthanised with Tricaine by the breeder. No experiments were carried out with live adult nor subadult fish before euthanasia. Accordingly, animal welfare guidelines were followed while zebrafish were kept by the breeder.

## 3 Results

**FIGURE 1:**
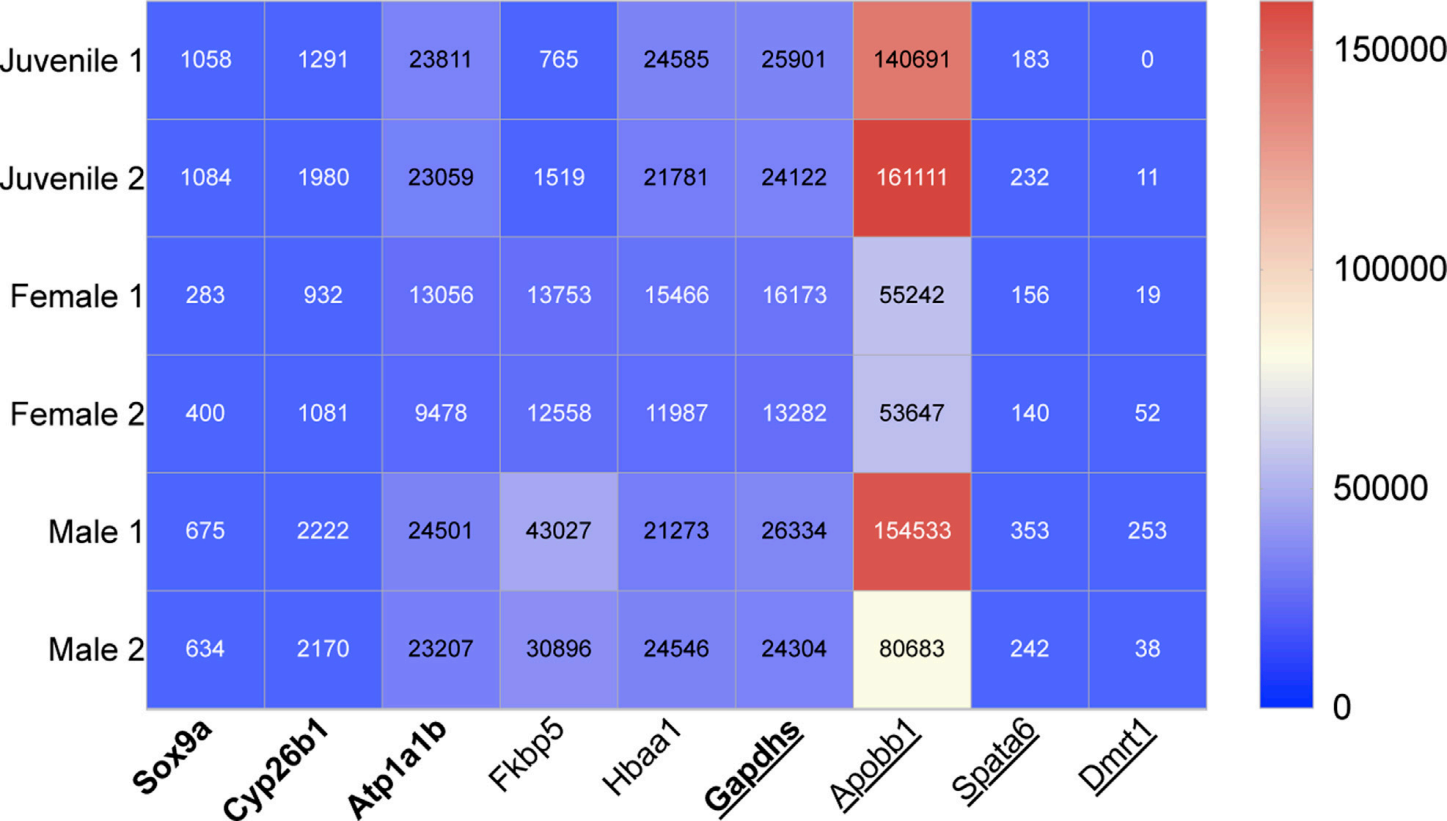
Heat map showing the relative transcript expression from NGS data of four previously identified male genes in bold letters (King et al., 2020) and five novel male genes, which can be used for early sex-determination in zebrafish (juveniles age 28dpf). Underscored no drug influence.

**FIGURE 2:**
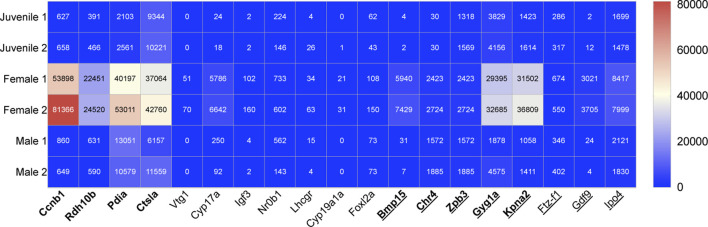
Heat map showing the relative transcript expression from NGS data of nine previously identified female genes in bold letters (King et al., 2020) and ten novel female genes, which can be used for early sex-determination in zebrafish (juveniles age 28dpf). Underscored no drug influence.

Five male and ten female genes which show great potential as novel early sex-determining gene markers were highlighted from zebrafish NGS data (Figures 1, 2, respectively). The 15 genes were put into three categories based on the results of previous published studies (Table 1). The three categories; category one: gene expression influenced by drugs, category two: drug exposure thought not to influence gene expression (sometimes it is unclear whether gene expression is influenced by drugs and more research is needed to clarify this) and category three: gene expression not influenced by drugs, were used to highlight if drugs have been previously found to affect gene expression of the selected early sex-determining genes.

**TABLE 1:** Summary of early-expressed sex-determining genes selected from a previous study and NGS data in this study and their relative category to indicate if their expression is influenced by drug exposure based on a literature review (category one: gene expression influenced by drugs, category two: drug exposure thought not to influence gene expression and category three: gene expression not influenced by drugs).

Gene	Sex determining gene	Category one	Category two	Category three	References for sex determining gene
Sox9a	Male	X	-	-	King et al. (2020)
Cyp26b1	Male	X	-	-	King et al. (2020)
Atp1b1a	Male	X	-	-	King et al. (2020)
Fkbp5	Male	X	-	-	NGS data from this study
Hbaa1	Male	X	-	-	NGS data from this study
Ccnb1	Female	X	-	-	King et al. (2020)
Rdh10b	Female	X	-	-	King et al. (2020)
Pdia	Female	X	-	-	King et al. (2020)
Ctsla	Female	X	-	-	King et al. (2020)
Vtg1	Female	X	-	-	NGS data from this study
Cyp17a1	Female	X	-	-	NGS data from this study
Igf3	Female	X	-	-	NGS data from this study
Nr0b1	Female	X	-	-	NGS data from this study
Lhcgr	Female	X	-	-	NGS data from this study
Cyp19a1a	Female	X	-	-	NGS data from this study
Foxl2a	Female	X	-	-	NGS data from this study
Gapdhs	Male	-	X	-	King et al. (2020)
Apobb1	Male	-	X	-	NGS data from this study
Spata6	Male	-	X	-	NGS data from this study
Dmrt1	Male	-	X	-	NGS data from this study
Bmp15	Female	-	X	-	King et al. (2020)
Chr4	Female	-	X	-	King et al. (2020)
Zpb3	Female	-	X	-	King et al. (2020)
Gyg1a	Female	-	X	-	King et al. (2020)
Kpna2	Female	-	X	-	King et al. (2020)
Ftz-f1	Female	-	X	-	NGS data from this study
Gdf9	Female	-	X	-	NGS data from this study
Ipo4	Female	-	X	-	NGS data from this study

The expression of a further four male (Sox9a, Gapdhs, atp1b1a and cyp26b1) and nine female genes (gyg1a, rdh10b, pdia, KPNA2, ccnb1, ctsla, Chr4, bmp15 and zbp3), which were previously identified as early-sex determining genes (King et al., 2020), were also compared to drug exposure and categorised into the three above influence categories (Figures 1, 2 respectively).

### 3.1 Genes selected for early male sex-determination

Five novel genes were selected from zebrafish NGS data; three of the five genes were among the list of transcripts with the highest differentiation between the males and females. The other two genes were selected from our NGS data because of highlighted sex differences in their expression. The following five sex-related genes were selected as potential markers of male sex-determination in juvenile zebrafish, 28dpf (Figure 1). These identifications are all supported by previous literature.

#### 3.1.1 FKBP prolyl isomerase 5 (fkbp5)

Based on NGS data, fkbp5 was expressed 2.8 times higher in males compared to females. Fkbp5 was also expressed in juveniles, 28dpf. The gene, fkbp5, was highlighted in the list of the top 13 male genes with the highest transcript differences between male and female zebrafish. The gene fkbp5 is involved in FK506 and heat shock protein binding, isomerase, peptidyl-prolyl cis-trans isomerase and peptidyl-prolyl cis-trans isomerase activity (Zfn.org—ZFIN ID: ZDB-GENE-030616-630).

#### 3.1.2 Apolipoprotein Bb, tandem duplicate 1 (apobb1)

Using NGS data, apobb1 was selected from the list of genes which showed the highest difference between male and female transcript data. There was also high expression of apobb1 in juvenile samples. On average adult male zebrafish expressed apobb1 2.1 times more than females based on transcript data. Apobb1 is predicted to have lipid transporter activity and is involved in cellular response to xenobiotic stimulus (Zfn.org—ZFIN ID: ZDB-GENE-030131-9732).

#### 3.1.3 Haemoglobin, alpha adult 1 (hbaa1)

Hbaa1 was selected from the table of gene expression which highlighted genes with the highest transcript count difference between males and females. Here, a high expression of hbaa1 was found in juveniles and adult male samples–transcript data of these was almost identical. Expression of hbaa1 was 1.7-fold higher in males compared to females based on NGS data. Hbaa1 is predicted to contribute to haptoglobin binding and peroxidase activity and is involved in response to hypoxia (Zfn.org—ZFIN ID: ZDB-GENE-980526-79).

#### 3.1.4 Double-sex and mab-3 related transcription factor 1 (dmrt1)

There was on average a four-fold higher expression of dmrt1 found in adult males compared with females, shown in NGS transcript data. Dmrt1 was also expressed weakly in juvenile NGS transcript data. It is predicted to have DNA-binding transcription factor activity; metal ion and sequence-specific DNA binding activity (Zfn.org—ZFIN ID: ZDB-GENE-050511-1).

#### 3.1.5 Spermatogenesis associated 6 (spata6)

NGS transcript data revealed an average of two times higher expression of spata6 in male compared to female samples. Expression of spata6 was also seen in juvenile samples of NGS data. Spata6 is predicted to have myosin light chain binding activity and to be involved in spermatogenesis (Zfn.org—ZFIN ID: ZDB-GENE-050417-351).

### 3.2 Genes selected for early female sex-determination

The following ten female genes were selected from our NGS data and with support from previous literature were highlighted as potential markers of female sex-determination in juvenile zebrafish, 28dpf (Figure 2).

#### 3.2.1 Vitellogenin 1 (vtg1)

We found from NGS data that there was a significantly higher female transcript expression of vtg1 compared with male zebrafish with on average 60-fold more transcripts for females compared to males. Based on NGS data, the expression of vtg1 is absent in both males and juveniles. Vtgs are female-specific, hepatically synthesised lipoproteins. They are the main carriers of retinoids and carotenoids (Zfn.org—ZFIN ID: ZDB-GENE-001201-1).

#### 3.2.2 Cytochrome P450, family 17, subfamily A, polypeptide 1 (cyp17a1)

Our data indicates a 36-fold increase in cyp17a in female zebrafish compared to males. Slight expression of cyp17a1 was also found in juvenile samples. Cyp17a1 is expressed in Leydig cells; brain; and gonad. Cyp17a1 exhibits 17-alpha, 20-alpha-dihydroxypregn-4-en-3-one dehydrogenase; 17-alpha-hydroxyprogesterone aldolase; and steroid 17-alpha-monooxygenase activity (Zfn.org—ZFIN ID: ZDB-GENE-040213-2).

#### 3.2.3 Cytochrome P450, family 19, subfamily A, polypeptide 1a (cyp19a1a)

Cyp19a1a was expressed on average 26 times higher in female compared to male samples based on our NGS transcript data. There is a lack of cyp19a1a in both male and juvenile samples based on NGS data. Cyp19a1a is predicted to have iron and haem-binding, as well as oxidoreductase activity. It is involved in several processes, including primary sex determination, germ-line fate; regulation of oestrogen biosynthetic process; and response to oestradiol (Zfn.org—ZFIN ID: ZDB-GENE-990415-43).

#### 3.2.4 Insulin-like growth factor 3 (igf3)

Using NGS data from zebrafish, we found that igf3 expression in females was on average 44 times that of males. Igf3 was weakly expressed in juvenile samples. Igf3 exhibits insulin-like growth factor receptor binding activity. It is involved in oocyte maturation and sperm capacitation and is localised to the extracellular space (Zfn.org—ZFIN ID: ZDB-GENE-080611-1).

#### 3.2.5 Nuclear receptor subfamily 5, group A, member 2 (ftz-f1, nr5a2 or ff1a)

Ftz-f1 gene was on average 1.6-fold higher expressed in females compared to males, based on NGS transcript data. Ftz-f1 was also expressed in juvenile zebrafish samples. The ftz-f1 gene exhibits DNA binding and DNA-binding transcription factor activity and is involved in several processes, including cartilage and digestive system development; and hepatoblast differentiation (Zfn.org—ZFIN ID: ZDB-GENE-990415-79).

#### 3.2.6 Growth differentiation factor 9 (gdf9)

NGS data from this study revealed, 240 times more gdf9 transcripts when adult zebrafish females and males were compared. Gdf9 was only slightly expressed in juvenile zebrafish based on transcript data. Gdf9 is predicted to have cytokine and transforming growth factor beta receptor binding activity (Zfn.org—ZFIN ID: ZDB-GENE-050221-7).

#### 3.2.7 Forkhead box L2a (foxl2a)

Foxl2a is on average 1.8 times higher expressed in female than in male samples. It was also expressed in juveniles, 28dpf. Foxl2a is predicted to have DNA-binding transcription factor and RNA polymerase II-specific activity. It is involved in maintenance of animal organ identity (Zfn.org—ZFIN ID: ZDB-GENE-060512-241).

#### 3.2.8 Nuclear receptor subfamily 0, group B, member 1 (nr0b1 or dax1)

Nr0b1 was on average 1.9 times more expressed in female than male zebrafish based on transcript data from this study. Expression of nr0b1 was also seen in juvenile transcript data. It is predicted to have transcription co-repressor and transcription factor binding activity and is involved in negative regulation of male gonad development and water homeostasis (Zfn.org—ZFIN ID: ZDB-GENE-070130-1).

#### 3.2.9 Importin 4 (ipo4)

Transcript data from NGS results indicated that ipo4 is on average expressed 4.2 times more in adult females when compared with adult males. NGS data in this study also showed expression of ipo4 in juvenile samples. Ipo4 is predicted to have nuclear import signal receptor and nuclear localisation sequence binding activity (Zfn.org—ZFIN ID: ZDB-GENE-041014-307).

#### 3.2.10 Luteinizing hormone/choriogonadotropin receptor (lhcgr)

Lhcgr was 5.1 times more expressed female zebrafish compared to males based on NGS transcript data. Based on our data, lhcgr was also shown to be expressed in juveniles, 28dpf. Gonadotropin receptor lhcgr exhibits luteinising hormone receptor activity and is involved in gamete generation and gonad development (Zfn.org—ZFIN ID: ZDB-GENE-040806-3).

## 4 Discussion

We assume from the gene expression patterns in NGS data that both juveniles are male (King et al., 2020), indicating that the sex was already genetically visible at this stage of development (28dpf). Although our data shows slight differences in transcript number across sex categories for adult danio, the transcript number across each sex is within the same range. The fact that both the juveniles are males explains why some of the highly expressed selected female genes showed no or very little expression in juveniles, namely, vtg1, cyp19a1a, igf3 and gdf9 (Figure 2). Despite the lack of expression in our NGS data, the expression of these four genes in juvenile and female zebrafish have previously been extensively studied and it is a well-established that these are early expressed female-determining genes. Hence why they were selected as early-sex determining markers for their comparison against drug exposure in this study. Studies referenced in this paper which were used to categorise gene expression change influenced by drug exposure include a range of model organisms. *Danio rerio* and humans as well as other species have orthologous genes which means these organisms can be used for gene expression comparison (Moyer et al., 2021).

The process of NGS is expensive (Schwarze et al., 2020) but it produces a huge amount of data, thus two samples per group were deemed sufficient in this study.

### 4.1 Category one: drug exposure influences expression of certain early sex-determining genes in *Danio rerio*

It is important to determine if the expression of previously discovered (King et al., 2020) and novel sex-determining genes is known to be affected by drug application. Certain drugs, including hormone or steroid-based drugs, are known to commonly affect the expression of female and male-specific genes. In mammals, oestrogen is the major female sex hormone that regulates secondary sex differentiation. Previous studies have shown that pharmacological manipulation of oestrogen production in zebrafish alters sex determination (Guiguen et al., 2010). It is not applicable to use genes which are affected by hormone levels as markers for revealing confounding sex differences in pharmaceutical trials.

#### 4.1.1 Formerly selected male sex-determining genes influenced by drug activity

##### 4.1.1.1 Sox9a

King et al. (2020) Sox9a is an early expressed, male sex-determining gene which plays a role in spermatogenesis and is predominantly expressed in the testis (Groh et al., 2011; Chen et al., 2017; Lin et al., 2017). A high expression of sox9a has been found in both zebrafish in males, juveniles, 28dpf and during earlier development (Jørgensen et al., 2008; King et al., 2020). Despite the fact that sox9a has strong potential as a candidate gene for early sex determination, previous studies involving long-term exposure to natural and synthetic P4 and norgestrel and other drugs affecting steroid levels have been shown to affect sox9a expression, thereby altering sex-determination of zebrafish. Tamoxifen is a selective oestrogen receptor modulator used to prevent and treat breast cancer. Exposure to tamoxifen during development induces abnormal upregulation of sox9 in sertoli cells of female mice which in turn causes testis differentiation (Yu et al., 2014). Therefore, its use as a sex-determining gene in pharmaceutical studies cannot be justified (van boxtel et al., 2010; Liang et al., 2015).

##### 4.1.1.2 Cyp26b1

Cyp26b1 is expressed highly in male and juvenile zebrafish (King et al., 2020). Retinoic acid (RA) is essential for spermatogenesis in male zebrafish. The relative level of RA in the gonads is correlated with sex-differentiation. Cyp26b1 degrades RA and has a higher expression in the testis. Cyp26b1 is upregulated in individuals of male fate. The loss of RA prevents developing gonads from entering into meiosis to form the ovaries, the opposite is true for female fated individuals (Rodríguez-Marí et al., 2013; Pradhan and Olsson, 2015). In mice, male transcript factors, sf1 and sox9 upregulate and activate cyp26b1. In gonads fated to become ovaries, foxl2 is highly expressed and acts an antagonist to cyp26b1 (Kashimada et al., 2011).

Retinoids have been well studied for their chemo-preventive properties. Retinoids are activated by their conversion to RA (Sedjo et al., 2004). In all vertebrates and some invertebrates, RA is a potent teratogen that, at pharmacological concentrations, can induce defects in congenital processes. RA signalling can be blocked by pharmaceuticals, thereby altering cyp26b1 level and sexual fate (Mark et al., 2006).

Anti-convulsants are a broad group of pharmacological agents which stabilise the level of nerve cell impulses and are used to treat epileptic seizures, bipolar disorder as well as for the treatment of neuropathic pain. Retinoids and vitamin A control have a vital role in sex-determination while the embryo is developing. Pharmaceuticals with anti-convulsant agents can have a huge effect on the retinoid metabolism. Due to the effect of cyp26b1 on RA expression during juvenile development, the altered retinoid metabolism by these drugs may significantly affect sex-determination (Nau et al., 1995). These results taken together indicate that cyp26b1 is not suitable to indicate sex variations during pharmaceutical trials.

##### 4.1.1.3 Atp1b1a

Atp1b1a is highly expressed in adult male, juvenile and developing zebrafish (Wang et al., 2008b; Hatzold et al., 2016; King et al., 2020). The oestrogen medication, Ethinylestradiol (EE) is commonly used in combination with progestins in birth control pills. EE has often been used to treat menopausal symptoms, gynaecological disorders, and certain hormone-sensitive cancers. Sewage effluent sometimes contains concentrations of EE2 which alters reproduction in fish. After zebrafish were exposed to EE2, atp1b1a was downregulated (Martyniuk et al., 2007).

In recent times, graphene oxide (GO) has been researched for application of drug delivery in order to discover its properties for multi-pharmaceutical action including those with photothermal therapy. GO has been found to be useful in cancer therapies. *In vitro* studies have shown that GO is a valuable nanomaterial for stem cell research in medicine as it has the potential to promote stem cell adhesion, growth and differentiation (Duran et al., 2015; Priyadarsini et al., 2018). After zebrafish were exposed to 100 μg/L GO, the expression of atp1b1a was vastly altered. GO exposure to zebrafish caused atp1b1a to be downregulated. With these points considered, atp1a1b is not suitable as a sex-determining marker due to the alteration in expression of this gene with application of oestrogen-based drugs or those containing GO (Zou et al., 2018).

#### 4.1.2 Novel male sex-determining genes influenced by drug activity

##### 4.1.2.1 Fkbp5

Fkbp5 gene is an important target of androgen signalling in the prostate. Androgen-deprivation leads to a decrease in FKBP51 and androgen stimulation resulting in up to 5-fold upregulation of FKBP5 (Amler et al., 2000). Therefore, FKBP5 is termed androgen-responsive, is conserved among species and plays a role in zebrafish male development (Magee et al., 2006). The transcript data shown in this study indicates juvenile expression of fkbp5 and an almost a 3-fold increase of fkbp5 gene expression in adult male zebrafish compared to females. This is in correlation with previous studies; therefore, it can be concluded that fkbp5 can be used as an early indicator of sex in zebrafish.

Fkbp5 and fkbp51 expression has been linked to stress response regulation and can be used in anti-depressant treatment. Fkbp5, the gene expressing the protein fkbp51 (FK506-binding protein 51), is a strong inhibitor of the glucocorticoid receptor (GR). Restoration of GR signalling has been found to be a treatment for depression. In a previous study, patients who responded to anti-depressant treatment had a significant reduction of fkbp5 gene and associated fkbp51 protein expression, whereas an increasing expression level of fkbp5 was observed in non-responders (Ising et al., 2019). Therefore, the use of fkbp5 as a gene for identifying sex related differences in drug trials cannot be justified.

##### 4.1.2.2 Hbaa1

In zebrafish, hbaa1 is expressed 19 organs including, eye; hematopoietic system; presumptive blood; and vasculature; with highest expression level in the spleen and 13th highest expression in the testis of zebrafish at fully formed stage. Zebrafish hbaa1 is orthologous to several human genes including HBZ (haemoglobin subunit zeta) (Zfn.org—ZFIN ID: ZDB-GENE-980526-79). Hbaa1 is expressed during zebrafish early development from zygote-1-cell-stage to adult-stage (Higano et al., 1997). Highest expression of hbaa1 in early development occurs at 35dpf (Tiedke et al., 2011). There are very few studies to our knowledge in which adult male and female zebrafish hbaa1 expression are compared. Our data indicates a higher expression of hbaa1 in adult male zebrafish compared with females as well as a high expression level in juveniles, therefore, hbaa1 should be considered as an early marker for male sex-determination in zebrafish but more research should be done to confirm this.

Again, few studies were found which show the effect of drugs on the expression of hbaa1 and its orthologs. Valproate (VPA) and its valproic acid, sodium valproate, and valproate semi-sodium forms are medications primarily used to treat epilepsy and bipolar disorder and prevent migraine headaches. A study found that VPA blocked the expression of human HBZ (Belrose et al., 2011; Gazon et al., 2016). More studies should now be carried out to investigate this pattern of expression in hbaa1 and its orthologs to discover for certain whether it can be discounted as a gene for uncovering sex-related differences during drug trials.

#### 4.1.3 Formerly selected female sex-determining genes influenced by drug activity

##### 4.1.3.1 Ccnb1

King et al. (2020) Ccnb1 has shown its potential to be an early expressed gene to indicate female sex (Yasuda et al., 2010; Horie and Kotani, 2016; King et al., 2020). The gene is expressed in the unfertilised egg, oocyte and mature ovarian follicle and involved in maturation and female sex determination in zebrafish (Kondo et al., 1997; Kondo et al., 2001; Knoll-Gellida et al., 2006; Wang et al., 2007; Nagahama and Yamashita, 2008; Kotani et al., 2013; Yasuda et al., 2013; Takahashi et al., 2014; Dingare et al., 2018; Takei et al., 2018; Yi et al., 2019).

Ccnb1 is significantly overexpressed in various cancer types including breast and prostate. A study showed that several tested drugs had an influence the expression of ccnb1; Doxorubicin, Cisplatin, Etoposide, Fluorouracil, Irinotecan, Ochratoxin A, Oxaliplatin and Tretinoin could decrease the expression of ccnb1, while Paclitaxel and Tamoxifen could increase the expression of ccnb1 (Ding et al., 2014). Additionally, resveratrol, a plant compound that acts like an antioxidant, has been used as an inhibitor of ccnb1 and can therefore regulate both the proliferation and apoptosis of pituitary tumour cells as well as altering the expression level of various EMT (epithelial-to-mesenchymal transition) markers (Li et al., 2019a). All things considered, the use of ccnb1 as an early sex determining gene in drug trials is not appropriate.

##### 4.1.3.2 Rdh10b

Rdh10b is more highly expressed in female and juveniles than in males (King et al., 2020). The gene is necessary for oxidisation of retinol to retinal and RA (Sandell et al., 2007). Retinoids are found in eggs and oocytes of marine and freshwater fish, teleosts, amphibians and mammals (Costaridis et al., 1996; Irie and Seki, 2002). RA is highly expressed in the gonad of female zebrafish and is necessary for sexual development in adult female but not adult male mice (Koubova et al., 2006; Belyaeva et al., 2020b). A decrease in RA results in a male phenotype (Rodríguez-Marí et al., 2013). In the ovary of zebrafish, genes which are necessary for the synthesis of retinal are upregulated (Levi et al., 2012). Insulin represses rdh10 expression by inactivating FoxO1 (Obrochta et al., 2015). The alteration rdh10b gene expression with insulin shows that it is not suitable as a gene to reveal the confounding variable of sex in FELS tests.

##### 4.1.3.3 Pdia

Pdia is expressed during zebrafish development. It is found highly expressed in the female gonad at juvenile and adult stages and been suggested as an early female sex-determining gene in zebrafish (Zheng et al., 2018; King et al., 2020). It has been observed that the expression of pdia decreases in the presence of tunicamycin, a mixture of homologous nucleoside antibiotics (Nair et al., 2007). Additionally, application of dithiothreitol (DTT), a reducing agent that causes the formation of unfolded proteins, causes the upregulation of pdia2 (Al-Sheikh et al., 2004). Due to the changing expression of pdia with drug influence its use as an early sex-determining gene for the discovery of drug related differences cannot be applied.

##### 4.1.3.4 Ctsla

Ctsla was previous selected as a female sex-determining gene (King et al., 2020). This maternally inherited gene is known to be involved in oogenesis and is highly expressed in juvenile zebrafish and the ovary and oocytes of adult zebrafish (Tingaud-Sequeira and Cerdà, 2007; Tingaud-Sequeira et al., 2011; King et al., 2020).

The mammalian ortholog of ctsla is ctsl. Nifedipine, a calcium antagonist, was shown to suppress ctsl activity and mRNA expression in mice (Nishimura et al., 2002). Previous studies have demonstrated that the expression of ctsl was upregulated in several cancers; therefore, supporting its use as a therapeutic target for cancer (Rudzińska et al., 2019). There is evidence that ctsl may be central in drug resistance development (Zheng et al., 2004). Further research has shown that by inhibiting ctsl, development of resistance to cancer drugs such as doxorubicin, a chemotherapy medication used to treat cancer, can be reversed, and prevented. No matter what level of resistance cancer cells have reached, their reaction to drugs is restored after ctsl inhibition (Zheng et al., 2009). Additionally, some anti-tuberculous and anti-leprotic drugs inhibit the lysosomal cysteine proteinases such as ctsl; therefore, changing the expression of the gene (Kamboj et al., 2003). These results taken together imply that ctsla is not suitable to indicate sex variations during drug trials.

#### 4.1.4 Novel female sex-determining genes influenced by drug activity

##### 4.1.4.1 Vtg1

Vtg1 exhibits antioxidant activity and is involved in cellular response to oestrogen, oestradiol, and xenobiotic stimulus. It is expressed in several structures, including the unfertilised egg and at various stages through early development; in zygote:1-cell-stage at 0hpf to 0.75hpf, at larval-stage, 5dpf and all the way to breeding adult-stage at 90dpf to 730dpf (Zfn.org—ZFIN ID: ZDB-GENE-001201-1). During vitellogenesis, vtg1 is secreted and transported to the ovaries in the plasma. NGS data in this study as well as previous research showed that vtg1 has a higher overall expression in females compared to males (Levi et al., 2012). Increased expression of vtg1 in females is essential for the maintenance of the female sex and the prevention of testis differentiation in zebrafish.

Observations from previous studies indicate that oestrogen, E2, stimulates gene expression of vtg1 (King et al., 2020). When juvenile zebrafish are exposed to the oestrogen 17alpha-ethynyl oestradiol, the sex ratio is significantly female-biased; in one case an entirely female population was produced after exposure (Örn et al., 2003). Therefore, the use of vtg1 would be unsuitable as a marker for identifying female zebrafish in FET with endocrine disrupting substances, since the gene is upregulated by oestrogenic substances (Leet et al., 2011). However, the evidence of juvenile expression of vtg1 from previous literature as well as the significant female transcript expression from NGS data displayed in this study, indicates the usefulness of vtg1 as an early precursor for sex-determination. For these reasons, vtg1 can be used as a molecular marker for the indication of feminisation but not for uncovering sex-related differences in drug discovery (Jin et al., 2009).

##### 4.1.4.2 Cyp17a1

Cyp17a1 is involved in androst-4-ene-3,17-dione biosynthetic and p4 metabolic process. Human orthologs of this gene are implicated in several diseases, including prostate cancer. In the zygote of zebrafish, cyp17a1 is already expressed during the 1-cell stage. Additionally, its expression is seen in the zygote at larval-stage, 5dpf; to breeding adult, 90dpf to 730dpf (Zfn.org—ZFIN ID: ZDB-GENE-040213-2). Elevated expression of cyp17a1 in female zebrafish can be seen in previous research; whereas cyp17a1a deficient zebrafish had male phenotypes (Zhai et al., 2018). A significantly higher expression of cyp17a1 can be seen in adult female zebrafish compared to males based on transcript data in this study and this is supported by previous research (Lin et al., 2017; Yan et al., 2017). These observations confirm that cyp17a1 is essential for female sexual development. It can be concluded from these findings that cyp17a1 could be suitable as a marker of female sex-specific expression in juvenile zebrafish.

Cyp17a1 has been found to be highly expressed in half about of human prostate carcinomas. Intracellular androgen is synthesised by these cancer cells. Abiraterone, a drug used to treat prostate cancer, can block nuclear accumulation of androgen receptors (ARs) which consequently suppresses expression of cyp17a1 (Giatromanolaki et al., 2019). The alteration of cyp17a1 gene expression with drug application means that it should not be selected to reveal sex-related differences with drug usage.

##### 4.1.4.3 Igf3

In zebrafish, Igf3 is expressed in several structures, including gill; gonad; head; kidney; and skeletal muscle; with fifth highest expression level in the female gonad at fully-formed stage (Zfn.org—ZFIN ID: ZDB-GENE-080611-1). Igf3 is expressed in early ovary development (Wang et al., 2008a; McMenamin et al., 2013) and its expression increases throughout the course of ovary differentiation (Li et al., 2011). Igf3 has been found to be expressed from zygote 1-cell-stage, 0.0 h–0.75h, to adult, 90days to 730 days (Zfn.org—ZFIN ID: ZDB-GENE-080611-1). Igf3 is generally thought of as a female biasing gene (Xia et al., 2018). The increased expression of igf3 in adult female zebrafish can be explained by its role in oocyte maturation (Li et al., 2011). Using transcript data from this study, an increased expression of igf3 can be seen in adult female zebrafish compared to males. Since the whole zebrafish was used for the analysis of gene expression and no tissue-specific expression analysis of the ovaries was performed, the eggs present in the females in our study possibly contributed to the increased expression of igf3. Despite this, analysis of igf3 reveals it as a potentially suitable gene for assigning a sex-specific expression profile in developing zebrafish.

Insulin-like growth factor 1 receptor (IGF-1R) has already been used as a therapeutic target (Dolgin, 2020). Studies have shown that the stable overexpression of igfbp3 could suppress the growth of tumour cells by inducing apoptosis and suppressing survival and growth of cells (Dar et al., 2010). Despite its use as an early genetic marker of sex-determination, due to the change in the expression of igf3 with drug application it cannot be used as a gene to reveal sex-related differences during drug trials.

##### 4.1.4.4 Nr0b1

Nr0b1 is predicted to have transcription corepressor activity and transcription factor binding activity. It is involved in negative regulation of male gonad development and water homeostasis. Nr0b1 is predicted to localise to the cytoplasm and nucleus and is expressed in several structures, including central nervous system; digestive system; fin; inter-renal primordium; and reproductive system, with highest levels of expression in the liver (Zhao et al., 2006). The gene is also expressed in earlier zebrafish development from gastrula-50%-epiboly-stage, 5.25hpf to 5.66hpf until adult-stage, 90dpf to 730dpf (Zfn.org—ZFIN ID: ZDB-GENE-070130-1). In zebrafish, nr0b1 mutants developed mostly as males (Chen et al., 2016). Mutations of the gene nr0b1 in zebrafish larvae, 13dpf, caused a decreased expression of the female-specific aromatase-encoding gene, cyp19a1a. This suggests that nr0b1 works during the early bipotential stage to promote female development (Chen et al., 2016).

Long-term oestradiol (E2) administration to the protandrous black porgy fish resulted in high levels of nr0b1 (Wu et al., 2008). Additionally, RA has been widely used to treat photoaged skin cancer and psoriasis (Lalli, 2014). Vitamin A is also crucial in activating Retinoic Acid Receptors (RAR). Both tretinoin and isotretinoin, are two synthetic forms of vitamin A. RA treatment has been found to increase the expression of nr0b1 (Nagl et al., 2009). These previous findings highlight changes in expression of nr0b1 after application of steroid-based and retinoid drugs, therefore, nr0b1 should not be used to indicate confounding sex identity with drug exposure (Li et al., 2016b).

##### 4.1.4.5 Lhcgr

Human orthologs of the gene, lhcgr, are implicated in leydig cell tumour; breast cancer; and gonadal disease. It is expressed in digestive system; gonad; and hypophysis from juvenile-stage; 45dpf to 89dpf through to adult-stage; 90dpf to 730dpf (Zfn.org—ZFIN ID: ZDB-GENE-040806-3). Recent studies have shown that lhcgr is more important in controlling female reproduction compared to its role in male development (Zhang et al., 2015; Song et al., 2020). In female, lhcgr plays a vital role in triggering final oocyte maturation and ovulation. Lhcgr, a follicle cell-specific marker is expressed in follicles and isolated follicle layers, interacts with female-specific, gdf9, and is thought to be essential for ovulation (Wang et al., 2005; Li et al., 2016a). Lhcgr encodes luteinizing hormone subunit beta, lhβ; the transcription levels of the pituitary glycoprotein hormone, lhβ, were upregulated in cyp17a1-deficient fish pituitary (Zhai et al., 2018).

Oestradiol (E2)-based drugs have been found to alter the expression of lhcgr. Use of 17β-oestradiol (E2), but not testosterone, changed the regulated the expression of lhcgr (Liu et al., 2011). Recent studies have shown that lhcgr is expressed at high levels in various gynaecological cancers and so it was predicted be a potentially important biomarker for cancer diagnosis and therapy, in particular for ovarian cancer (Xiong et al., 2019; Zhong et al., 2019). FSH receptor binding inhibitor (FRBI) suppresses the expression of lhcgr mRNA and protein in sheep cumulus-oocyte complexes (COCs) (Wei et al., 2018). The mechanism of FRBI as an antagonist of lhcgr also has potential in aiding ovarian diseases and ovarian and follicular functioning in order to promote fertility in humans and animals (Wei et al., 2017; Lai et al., 2018). Although lhcgr has been identified as a potential drug target for cancer therapy and methods to apply this have been refined, there have so far been very few studies showing how these anti-cancer drugs affect the expression of lhcgr (Cavalli et al., 2016; Gao et al., 2018). Further studies of lhcgr expression levels in relation to drug application are needed to reveal if it can be used as an early expressed gene for unravelling confounding sex variables during anti-cancer drug development.

##### 4.1.4.6 Cyp19a1a

Cyp19a1a is expressed in brain; eye; female organism; the ovary, ovarian granulosa cells; and the reproductive system; with highest expression level in female gonad (Chiang et al., 2001). The expression of the gene begins at zygote-1-cell-stage, through to adulthood (Zfn.org—ZFIN ID: ZDB-GENE-990415-43). Cyp19a1a is expressed at the highest level in the female gonad at juvenile stage, 30 to 44 days. It is also expressed in the immature gonad, at larval stage, 14 to 20dpf; in the gonad and female organism at larval stage, 21 to 44dpf; and in the mature ovarian follicle at fully-formed stage (Santos et al., 2007; Yu et al., 2014; Valdivieso et al., 2019). Cyp19a1a is a female promoting gene; the importance of cyp19a1a in the process of female sex differentiation was demonstrated when the silencing of the gene produced only male offspring (Lau et al., 2016; Yin et al., 2017). Other studies complement these findings whereby cyp19a1a is stated to be a female sex-determining gene (Santos et al., 2007; Jørgensen et al., 2008; Tong et al., 2010a; Rodríguez-Marí et al., 2010; Chen et al., 2017; Crowder et al., 2018b; Valdivieso et al., 2019) The NGS transcript data described here also complies with these findings. Taken together this implies that cyp19a1a could be used as an earlier genetic marker for indicating female sex-determination.

The expression of cyp19a1a in the gonad of labeo rohita fry changed after treated with fadrozole, an aromatase inhibitor. Equally treatment with 17β-oestradiol (E2) had no effect on the expression of cyp19a1a However, other studies have shown that two endocrine disrupting chemicals (EDCs) - an exogenous oestrogen 17β-oestradiol (E2) and the oestrogen mimic 4-n-nonylphenol (NP) cause a significant reduction in the expression of cyp19a1a in ovarian tissues with complete inhibition at the higher concentrations; therefore, implying that drugs with these actions can affect the expression of cyp19a1a (Shanthanagouda et al., 2013). More studies should be carried out to clarify the effect of different drugs on the expression of cyp19a1a, so that its use as an early sex-determining gene for uncovering sex-related difference with drug application can be definitively stated.

##### 4.1.4.7 Foxl2a

Foxl2a is expressed in female zebrafish; in the gonad; ovarian follicle and ovary at larval-stage, 14dpf to 20dpf until breeding adult, 90dpf to 730dpf (Cocquet et al., 2002; Uchida et al., 2002; Tong et al., 2010a). Our data also shows adult female and juvenile, 28dpf, expression of foxl2a in zebrafish. Foxl2a in combination with other genes is essential to regulate zebrafish hormone producing ovarian cells and is necessary for ovary development, growth, maintenance and folliculogenesis; its loss significantly affects female development (Selman et al., 1993; Uchida et al., 2002; Cocquet et al., 2003; Tong et al., 2010b; Herpin and Schartl, 2015; Kossack and Draper, 2016). This indicates the importance of foxl2a in preventing female-to-male sex reversal by repressing testis determining genes, dmrt1 and amh (Uchida et al., 2002; Uhlenhaut et al., 2009; Matson et al., 2011; Schlessinger et al., 2011; Huang et al., 2017; Yang et al., 2017; Crowder et al., 2018b). Based on transcript data in this study, a 1.8-fold increase of foxl2a gene expression was seen in adult female zebrafish compared to males. This provides valid evidence for its use as an early female gene marker in zebrafish.

Mutations in foxl2a have been found to cause several diseases; blepharophimosis syndrome (BPES), a complex eyelid malformation associated with premature ovarian failure, ovarian dysfunction–polycystic ovary syndrome (PCOS) and granulosa cell tumours and keloid scars (Cocquet et al., 2002; Fleming et al., 2010; Verdin and De Baere, 2012). Regulation of expression of foxl2 or its downstream targets with drugs may provide a potential target for therapy of these diseases but these must first be tested for clarification of this (Verdin and De Baere, 2012). Studies have shown that after treatment with oestradiol and 2-methyl-testosterone, foxl2 was upregulated and downregulated, respectively (Jiang et al., 2011). This suggests that foxl2 would be affected by steroid-based drug application and therefore, not suitable for uncovering sex-related variables to steroid-based drug treatment (Wu et al., 2019).

### 4.2 Category two: Drug exposure thought not to influence expression of certain previously discovered early sex-determining genes in *Danio rerio*–More research needed

In literature, it was unclear whether certain sex-determining genes are affected by drug usage. In some cases, previous studies could be found which identify drugs which affect sex determining gene expression levels and in other studies the same genes were identified as potential therapeutic targets, but no studies could be found which link these and indicate that drugs directly alter the expression of these gene. Therefore, further investigation into their expression level with exposure to various drugs is needed to clarify their use in pharmaceutical trials to reveal confounding sex-related differences.

#### 4.2.1 Formerly selected male sex-determining genes not influenced by drug activity

##### 4.2.1.1 Gapdhs

King et al. (2020) The gapdhs gene encodes a protein and the testis-specific enzyme, glyceraldehyde-3-phosphate dehydrogenase spermatogenic, gapdhs. They are vital for energy production, sperm motility to ensure male fertility (Welch et al., 1995; Kuravsky et al., 2011). Gapdhs is expressed in adult male zebrafish as well as during development and in juveniles (Liu et al., 2013; King et al., 2020). This potential target is known to belong to a well-known druggable protein family which implies that gapdhs can be used as an early sex determining gene without its expression level being affected by drug application (Gottwald et al., 2006). More research is needed to clarify this finding.

#### 4.2.2 Novel male sex-determining genes not influenced by drug activity

##### 4.2.2.1 Apobb1

Apobb1 is expressed in intestine; liver; and yolk syncytial layer. Expression occurs in early development from gastrula 50% epiboly-stage and continues to adulthood (Otis et al., 2015). Apobb1 is expressed at lower levels with vitellogenin and not expressed in the ovary (Levi et al., 2012). There is a lack of studies regarding expression levels of apobb1 comparing adult male and female zebrafish. Our data shows on average more than a two-fold increase in transcripts of male zebrafish compared to female zebrafish as well as expression in juvenile. These findings give reason for the selection of apobb1 as a potential early male sex-determining gene. No previous literature indicates that drug activity influences apobb1 expression levels, further studies should also be carried out to prove this in order to highlight apobb1 as an early male sex-determining gene for uncovering sex-related variables with drug application.

##### 4.2.2.2 Spata6

In mice and zebrafish, spata6 is predicted to localise to the sperm connecting piece during spermiogenesis. The sperm connecting piece is essential for linking the developing flagellum to the head in late spermiogenesis. When the expression of spata6 is disrupted males are sterile; this is due to the disruption to the formation of the sperm connecting piece which leads to acephalic spermatozoa in both the epididymis and ejaculates (Yuan et al., 2015; Sujit et al., 2020). In zebrafish, spata6 is expressed in neural tube; spinal cord; and ventral mesoderm, spermatids and mature spermatozoa (Yuan et al., 2015). Spata6 is expressed from 20dpf and increases to adulthood (Oh et al., 2003). Spata6 is a known pro-male gene and was upregulated in the ovaries of fish living in high-population density groups, showing stress from environmental factors causes masculinisation of zebrafish populations (Valdivieso et al., 2019). Previous literature taken with our NGS results show spata6 to be an early male sex-determining gene.

Spata6 is thought to play a key role in testicular germ cell tumours (TGCTs). Downregulation of spata6 results in apoptosis of TGCTs (Huo et al., 2015). This indicates that spata6 could be used as a potential gene target for drug therapy. However, more studies are needed to prove or disprove this and therefore indicate whether it can be used when reveal confounding sex variables during drug trials.

##### 4.2.2.3 Dmrt1

Dmrt1 is involved in gamete generation; male gonad development; and male sex. Dmrt1, which acts downstream of male SYR, has previously been shown to be more strongly expressed in males than in females (Jørgensen et al., 2008; Chen et al., 2017). It is known to maintain the male fate and control gonadal differentiation in vertebrate and non-vertebrate species (Matson et al., 2011; King et al., 2020). This is supported by evidence of higher expression of dmrt1 in male when compared with female zebrafish in NGS data from this study. It is expressed in 14 organs with highest expression level in testis (fully formed stage) of zebrafish. The expression of dmrt1 was also found to be expressed in juveniles throughout development; it is highly expressed in blastula stage of zebrafish (3.33h–3.66 h) through to breeding adult stage (90d-730 d) (Jørgensen et al., 2008).

Dmrt1 is thought to have an influence on sex-specific patterns of childhood asthma, and its expression in testis tissue suggests a potential involvement in hormone regulation (Schieck et al., 2016). It may therefore act as a potential drug target gene for asthma suffers and steroid-based genes may influence its expression. If drugs are found which change the expression of dmrt1 for therapeutic reasons, its use in human drug development as an early sex-determining gene for identification of sex-related difference is not appropriate.

#### 4.2.3 Formerly selected female sex-determining genes not influenced by drug activity

##### 4.2.3.1 Bmp15

King et al. (2020) Bmp15 is expressed in the gonads of juvenile and adult female zebrafish as well as in the ovaries of humans (Fitzpatrick et al., 1998; Paulini and Melo, 2011; King et al., 2020). It negatively regulates oocyte development, function and maturation through its own activity as well as by increasing expression of other genes which convert androgens to oestrogens, namely, cyp19a1a or decreasing the expression of the male determining gene, dmrt1 (Otsuka et al., 2000; Clelland et al., 2006; Matson et al., 2011; Yan et al., 2017; Hosseini et al., 2019). Loss of bmp15 in adult zebrafish results in a female-to-male sex reversal (Dranow et al., 2016; Kossack and Draper, 2016; Crowder et al., 2018a). In Humans, loss of bmp15 leads to disruption in ovary function (Galloway et al., 2000; Di Pasquale et al., 2004). Drug application has not been previously shown to affect bmp15 expression and so its use as a female sex-identifying gene in drug discovery could be warranted. More tests are needed to prove that bmp15 expression is not affected by drug application.

##### 4.2.3.2 Chr4

Chr4 expression has previously been linked to female sex determination in zebrafish and is expressed in juvenile stages (Charlesworth et al., 2005; Anderson et al., 2012; Howe et al., 2013; King et al., 2020). After research was carried out to discover changes in expression of chr4 with drug application, it was found that there were no previous studies which indicate that expression of chr4 is affected by drug application. Further studies should be carried out to clarify this result and its potential use for uncovering sex-related variables during drug trials.

##### 4.2.3.3 Zpb3

Zpb3 gene expression is partly influenced by folliculogenesis-specific basic helix-loop-helix, Fig alpha, Fig α, which is needed in female development (Zfn.org—ZFIN ID: ZDB-GENE-031121-1, King et al., 2020). The germ cell-specific transcription factor, Fig α has been found in mammals and zebrafish where it is necessary for ovary differentiation and preservation (Soyal et al., 2000; Kanamori et al., 2006; Jørgensen et al., 2008; Schlessinger et al., 2011). As previously shown zpb3 is more highly expressed in oocytes and can be used as an early marker for female (Onichtchouk et al., 2003). No previous literature indicates that drug activity influences zpb3 expression levels. Further studies are needed to indicate the use of zpb3 to reveal sex differences with drug exposure during pharmaceutical trials.

##### 4.2.3.4 Gyg1a

Gyg1a is highly expressed in developing, juvenile and female adult zebrafish (Maanen et al., 1999; Wen et al., 2005; King et al., 2020). No previous study has provided evidence that drugs can influence the expression of gyg1a, more research is needed to clarify this finding.

##### 4.2.3.5 Kpna2

Kpna2 is highly expressed in developing and adult female zebrafish gonads and its use as an early female sex-determining gene has been previously discussed (Major et al., 2011; Cui et al., 2020; King et al., 2020). Kpna2 overexpression has been linked to various human cancers, including, breast, ovarian endodermal sinus and ovarian primitive germ cell tumours (Huang et al., 2018; Christiansen and Dyrskjøt, 2020). An inhibitor of kpna2 could in theory act as a prospective drug to reduce growth of cancer cells. The genes, IRF1 has been shown to negatively regulate the expression of kpna2. Therefore, drugs which target IRF1 gene could disrupt normal kpna2 expression. Additionally, some studies have shown that kpna2 may be dependent on p53 nuclear translocation to support autophagy in order to allow chemoresistance and metastasis of certain cancerous cells, OSCC (Oral squamous cell carcinoma) (Lin et al., 2018). Although the use of kpna2 as a therapeutic target has been previously identified, studies regarding the relative gene expression levels with drug application are lacking and are required before kpna2 can be used as an early sex-determining gene for uncovering sex differences during drug trials.

#### 4.2.4 Novel female sex-determining genes not influenced by drug activity

##### 4.2.4.1 Ftz-f1

Ftz-f1 is expressed in several structures, including digestive system; endocrine system; head; nervous system; and slow muscle cell. It is expressed from zygote:1-cell-stage, 0.0h–0.75 h until breeding-adult-stage, 90days to 730 days (Tong et al., 2010b) (Zfn.org—ZFIN ID: ZDB-GENE-990415-79). At around 12dpf, when female germ cells start to rapidly grow, the initial sign of sexual dimorphism occurs. At this time somatic cells start to increase expression of ftz-f1 transcription factors (Tong et al., 2010b). Ftz-f1 has been previously associated with female sex-determination in zebrafish (Cao et al., 2012). The zebrafish transcript data which is presented here showed an increase of 1.6-fold in females compared to males. Ftz-f1 can therefore, be considered for identification of female zebrafish early in development.

Overexpression of ftz-f1 enhances activation, implicating ftz-f1 as a critical component of the juvenile hormone response (Dubrovsky et al., 2011). Additionally, amino acid sequencing of ftz-f1 gene revealed that the gene protein is a member of the nuclear hormone receptor superfamily (Lavorgna et al., 1991). This research could therefore imply that the expression of ftz-f1 can be altered by hormone-based drugs. However, no previous studies revealed that ftz-f1 expression was altered by drug application and more research should be carried into this before it can be classified as an earlier sex-determining gene for use in revealing sex-related variables occurring from use of drugs.

##### 4.2.4.2 gdf9

Gdf9 is an oocyte specific factor encoding proteins which are secreted by oocytes (Clelland et al., 2006; Paulini and Melo, 2011). It is involved in juvenile and adult female sex-determination in zebrafish and is essential for ovarian follicle development in mammals (Silva et al., 2005; Clelland et al., 2006; Gilchrist et al., 2008; Oron et al., 2010; Chen et al., 2017; Strauss and Williams, 2019). It is expressed in fertilised egg; gonad; and immature gonad (Fitzpatrick et al., 1998). The expression of gdf9 starts in the gonads before sex differentiation, and its expression dramatically increases in the differentiated ovary, with a similar expression pattern to that of cyp19a1a. Gdf9 significantly suppresses the expression of male amh while increasing that of activin beta subunits, inhbaa and inhbb. Knock-down expression of gdf9 creates a male-biased sex ratio. Gdf9 promotes follicle-stimulating hormone (FSH)-induced P4 production and STAR expression (Leitão et al., 2014; Li et al., 2019b). P4 is an endogenous steroid and progestogen sex hormone involved in the menstrual cycle, pregnancy, and embryogenesis of humans and other species (Chen et al., 2017). Our transcript data along with previous knowledge supplies evidence for gdf9 as an early gene marker in zebrafish sex-determination.

In humans, gdf9 was recently shown to be a regulator of the development and spread of certain cancer cells including those of the breast and kidney. Gdf9 expression is decreased in clear-cell renal cell carcinoma (CCRCC) which is linked to long-term survival of patients. Although this indicates that gdf9 is a potential tumour suppressor in CCRCC which can be used a potential target for tumour drug development, very few studies have looked at drugs which target the gene for therapeutic reasons nor have they indicated the relative level of gdf9 expression (Hanavadi et al., 2007; Du et al., 2012; Du et al., 2014).

##### 4.2.4.3 Ipo4

Ipo4 has a role in protein import into nucleus. It is expressed in 33 zebrafish organs with forth highest expression in the female gonad and 14th highest expression in the mature ovarian follicle [bgee. org—gene: ipo4— ENSDARG00000041493—Danio rerio (zebrafish)]. Ipo4 is expressed during development from 0dpf until 56dpf, peaking in expression level at 30dpf (Major et al., 2011). In this study, adult female zebrafish transcript data for ipo4 is around four times higher in adult females when compared to the adult males indicating it as a potential marker for early female sex-determination.

Ipo4 was found to be downregulated in both SARS-Cov-2 infection and aging (Belyaeva et al., 2020a). It could, therefore, provide a target for drug therapy for these. Ipo4 is thought to be a key gene contributing to the progression and development of gastric and other cancers (Xu et al., 2019; Zhou et al., 2020). The drugs, guanosine triphosphate and mkc-1 both have both been linked to the gene ipo4. Mkc-1 has an antineoplastic agent, meaning it inhibits the growth and spread of tumours or malignant cells (https://www.genecards.org/cgi-bin/carddisp.pl?gene=IPO4). This also highlights ipo4 as a gene target for cancer drug therapy. However, more studies are also needed for clarification of the action of these drugs on the expression of ipo4.

### 4.3 Category three: Drug exposure does not influence expression of certain previously discovered early sex-determining genes in *Danio rerio*–Evidence exists already

None of the previously selected early sex-determining genes (King et al., 2020) as well as none of the additional genes highlighted in this study were found to not be influenced by drug usage. This reveals the need for research to be focused in this area. Genes in which previous studies show that their expression is not influenced by drug exposure would have great potential to be used as candidate genes for revealing confounding sex variables in drug discovery. The genes highlighted in this study which show potential to be used for revealing sex variables in pharmaceutics trials (see category two) should now be evaluated further to clarify which of the two categories (category one or category three) they can be placed into.

## 5 Conclusion

We reveal here several male and female marker genes in early zebrafish development which are not known to be disrupted during drug exposure. These show great potential to unlock the confounding variable of sex in order to reveal sex-related differences during drug trials. These genes should be tested in the near future to guarantee their usefulness within drug development and to show if the effect on the molecular level would lead to a skewed sex ratio in environmental toxicity studies (Zhang et al., 2013). Results from NGS data and the literature review clearly confirm the differences in sensitivity within a species and indicate that females are more susceptible to drug exposure than males (Chen et al., 2021).

We uncover that sex-related genes are very often affected in some way by drugs. From this we deduce that there will be a sex-based difference in susceptibility to drugs, so we emphasise the importance of producing males and female-specific pharmaceuticals at specific sex doses. The model of the healthy male volunteer in pharmaceutical studies should be re-thought to allow safe dosage of drugs to prescribed for females. Drugs interfere with hormones and the gene expression pathway, likewise, hormones and genes influence drug metabolism. So, while it can be said that the sex of an individual is confounding the drug, likewise the drug is also confounding the sex.

Our findings emphasise the importance of promoting sex-specific drugs which would positively affect human pharmaceutical drug discovery. This paper paves the way in connecting the vital ‘missing link’ between juvenile sex-related genes and drug response which will greatly impact the future of pharmaceutical trials.

## Data Availability

The data discussed in this publication have been deposited in NCBI’s Gene Expression Omnibus (Edgar et al., 2002) and are accessible through GEO Series accession number GSE234104 (https://www.ncbi.nlm.nih.gov/geo/query/acc.cgi?acc=GSE234104).
